# Gold-Derived Molecules as New Antimicrobial Agents

**DOI:** 10.3389/fmicb.2022.846959

**Published:** 2022-03-23

**Authors:** Carlos Ratia, Raquel G. Soengas, Sara M. Soto

**Affiliations:** ^1^ISGlobal, Hospital Clínic—Universitat de Barcelona, Barcelona, Spain; ^2^Departamento de Química Orgánica e Inorgánica, Universidad de Oviedo, Oviedo, Spain

**Keywords:** gold(I) complexes, gold(III) complexes, antibacterial, mechanism of action, toxicity

## Abstract

Antimicrobial resistance is considered one of the three most important health problems by the World Health Organization. The emergence and spread of an increasing number of antimicrobial-resistant microorganisms make this a worldwide problem. Antibiotic-resistant bacteria are estimated to be the cause of 33,000 deaths in Europe and 700,000 worldwide each year. It is estimated that in 2050 bacterial infections will cause 10 million deaths across the globe. This problem is concomitant with a decrease in the investment and, therefore, the discovery and marketing of new antibiotics. Recently, there have been tremendous efforts to find new effective antimicrobial agents. Gold complexes, with their broad-spectrum antimicrobial activities and unique modes of action, are particularly relevant among several families of derivatives that have been investigated. This mini review revises the role of gold-derived molecules as antibacterial agents.

## Introduction

In spite of the important role of antibiotics in human health during the 60s and 70s, infectious diseases continue being the second cause of death worldwide (Spellberg et al., 2004). The emergence and spread of drug-resistant microorganisms (bacteria, fungi, virus, and parasites) are among the most important health threats today. This issue is especially alarming regarding antibiotic resistance in bacteria, as the rapid spread of multi- and pan-resistant bacteria in hospitals and in the community is associated with an increase in mortality and morbidity. It has been estimated that if no measure is taken in the next years, the number of deaths by infections caused by resistant bacteria could increase up to 10 million each year, exceeding the 1.8 million deaths by cancer. However, a recent study reported that infections associated with antimicrobial resistance are the first cause of death worldwide surpassing those caused by cancer, with 4.95 million of deaths in 2019 (Antimicrobial Resistance Collaborators, 2022). At an economic level, the infections by resistant bacteria represent 2.5 million days of extra hospitalization per year only in the European Union, with a cost of about 900 million euros (Glasner et al., 2013).

Following the discovery of cephalosporins in 1968, the discovery of new antibiotics with new mechanisms of action has slowed (Powers, 2004). After that, most of the antibiotics developed were derivatives from the existing classes and are considered as “new-generation” antibiotics. However, the use of new antibiotics has, sooner or later, been followed by the emergence of bacterial strains resistant to these antibiotics (Saga and Yamaguchi, 2009).

The decrease in investment for the search for antibiotics by pharmaceutical companies could be due to the time-consuming and financially taxing venture that traditional methods employed to discover new antibiotics require. This has led researchers to mine existing libraries of clinical molecules in order to repurpose old drugs for different clinical diseases as antimicrobials. This effort led to the discovery of the gold complex auranofin, a drug initially approved as an anti-rheumatic agent, which also possesses potent antibacterial activity in a clinically achievable range (Cassetta et al., 2014). Interest in antimicrobial gold complexes is not new. The work of Robert Koch at the end of the 19th century demonstrated that potassium dicyanidoaurate(I), K[Au(CN)_2_], showed activity against *Mycobacterium tuberculosis*, a causative agent of tuberculosis (Higby, 1982). Since then, interest in the anti-infective activity of gold complexes has prompted the evaluation of a large number of derivatives as potential agents against a high number of bacteria, fungi and parasite species.

So far, most studies have been focused on gold(I) complexes in connection with the remarkable bactericidal effects of Au(I) phosphine complex auranofin. By contrast, although gold(III) complexes have been intensively investigated as antitumor agents due to their similarities with platinum(II) (Nardón et al., 2014), until recently there were very few studies related to their antimicrobial activity (Glišić and Djuran, 2014). However, the serious threat of resistant “super-bugs” has led to the evaluation of these antitumor drugs also as antimicrobials.

In the present work, the antibacterial activity of both gold(I) and gold(III) representative complexes is revised.

## Gold(I) Complexes

**FIGURE 1:**
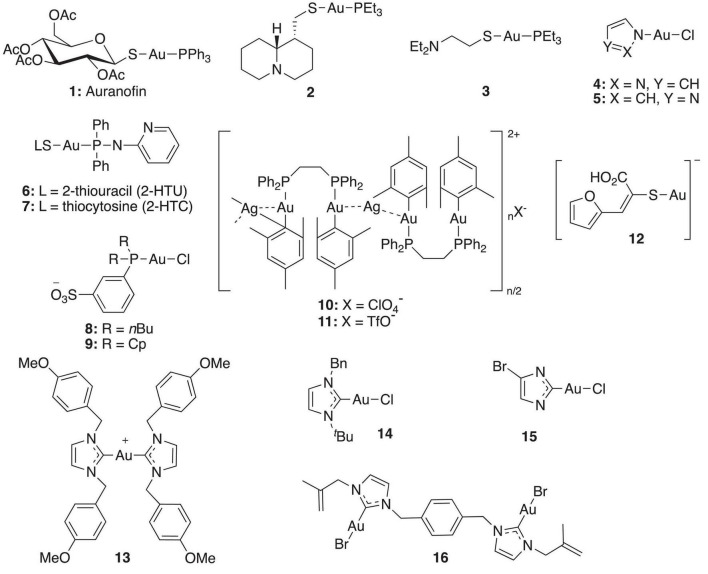
Most representative gold(I) complexes with antimicrobial activity.

In recent years, gold(I) compounds have been considered of clinical relevance due to their anti-rheumatic activity as well as their anticancer properties. In addition, the interest in these compounds as antibacterial agents is increasing, and different families of gold(I) have been investigated, including the Au(I) N-heterocyclic carbenes (NHCs) (Özdemir et al., 2004). A selection of gold(I) complexes with antimicrobial activity and their chemical structure is shown in Figure 1, as well as a compilation of their antibacterial (Table 1), antifungal (Table 2) and antibiofilm activities (Table 3).

**TABLE 1:** Antibacterial activity (MIC) of the gold(I) complexes.

Compound	MIC (μ g/mL)								References
	Gram-positive						Gram-negative		
	*S. aureus*	*E. faecalis*	*B. subtilis*	*B. cereus*	*S. epidermidis*	*E. faecium*	*E. coli*	*P. aeruginosa*	
Auranofin	0.125–0.25	n.r.	n.r.	n.r.	n.r.	n.r.	31.25	>250	Cassetta et al., 2014
**2**	0.12	n.r.	n.r.	n.r.	n.r.	n.r.	15.63	>250	Novelli et al., 1999
**3**	0.12–0.24	n.r.	n.r.	n.r.	n.r.	n.r.	7.81–15.63	>250	Novelli et al., 1999
**4**	7.9	n.r.	15.7	n.r.	n.r.	n.r.	>1000	>1000	Nomiya et al., 2000
**5**	125	n.r.	125	n.r.	n.r.	n.r.	>1000	>1000	Nomiya et al., 2000
**6**	3	3	n.r.	n.r.	n.r.	n.r.	>60	n.r.	Fillat et al., 2011
**7**	3	3	n.r.	n.r.	n.r.	n.r.	18	n.r.	Fillat et al., 2011
**8**	1*^a^*	n.r.	n.r.	1*^a^*	n.r.	n.r.	100*^a^*	n.r.	Elie et al., 2009
**9**	10*^a^*	n.r.	n.r.	10*^a^*	n.r.	n.r.	100*^a^*	n.r.	Elie et al., 2009
**10**	10	n.r.	n.r.	1	n.r.	n.r.	10	n.r.	Frik et al., 2012
**11**	10	n.r.	n.r.	10	n.r.	n.r.	10	n.r.	Frik et al., 2012
**12**	<6.25	n.r.	<6.25	n.r.	n.r.	n.r.	100	100	Barreiro et al., 2012
**13**	3.12	3.12	n.r.	n.r.	6.25	n.r.	n.r.	3.12	Özdemir et al., 2004
**14**	n.r.	n.r.	6.69	n.r.	n.r.	n.r.	>1000	>1000	Ray et al., 2007
**15**	0.24	n.r.	n.r.	n.r.	n.r.	1.18	n.r.	n.r.	Schmidt et al., 2017
**16**	3.51	n.r.	n.r.	n.r.	n.r.	n.r.	n.r.	1.75	Samanta et al., 2013

*n.r., Not reported; ^a^Minimum concentration to create a clearing zone of 0.5 cm: 1, 10, 100.*

**TABLE 2:** Antifungal activity (MIC) of the gold(I) complexes.

Compound	MIC (μ g/mL)							References
	*C. albicans*	*C. glabrata*	*C. parapsilosis*	*C. neoformans*	*A. fumigatus*	*A. niger*	*S. cerevisae*	
Auranofin	0.25–1	1–16	1–16	1–2	n.r.	n.r.	n.r.	Wiederhold et al., 2017
**2**	7.81	n.r.	n.r.	n.r.	n.r.	15.63	n.r.	Novelli et al., 1999
**3**	125	n.r.	n.r.	n.r.	n.r.	31.25	n.r.	Novelli et al., 1999
**4**	250	n.r.	n.r.	n.r.	n.r.	>1000	500	Nomiya et al., 2000
**5**	500	n.r.	n.r.	n.r.	n.r.	>1000	>1000	Nomiya et al., 2000
**6**	n.r.	n.r.	n.r.	n.r.	n.r.	n.r.	n.r.	Fillat et al., 2011
**7**	n.r.	n.r.	n.r.	n.r.	n.r.	n.r.	n.r.	Fillat et al., 2011
**8**	n.r.	n.r.	n.r.	n.r.	n.r.	n.r.	100*^a^*	Elie et al., 2009
**9**	n.r.	n.r.	n.r.	n.r.	n.r.	n.r.	100*^a^*	Elie et al., 2009
**10**	n.r.	n.r.	n.r.	n.r.	n.r.	n.r.	>100	Frik et al., 2012
**11**	n.r.	n.r.	n.r.	n.r.	n.r.	n.r.	>100	Frik et al., 2012
**12**	n.r.	n.r.	n.r.	n.r.	n.r.	n.r.	n.r.	Barreiro et al., 2012
**13**	200	n.r.	n.r.	n.r.	n.r.	n.r.	n.r.	Özdemir et al., 2004
**14**	n.r.	n.r.	n.r.	n.r.	n.r.	n.r.	n.r.	Ray et al., 2007
**15**	n.r.	n.r.	n.r.	n.r.	n.r.	n.r.	n.r.	Schmidt et al., 2017
**16**	3.51	n.r.	n.r.	n.r.	1.75	n.r.	n.r.	Samanta et al., 2013

*n.r., Not reported; ^a^Minimum concentration to create a clearing zone of 0.5 cm: 1, 10, 100.*

**TABLE 3:** Antibiofilm activity of the gold(I) and gold(III) complexes.

Compound	Concentration (μ g/mL)								References
	Gram-positive					Gram-negative			
	*S. aureus*	*S. haemolyticus*	*B. subtilis*	*K. pneumoniae*	*E. faecium*	*E. coli*	*P. aeruginosa*	*A. baumannii*	
**Gold(I)**									
Auranofin	7.99*^ a^*	n.r.	n.r.	n.r.	n.r.	n.r.	6.24*^a^*	n.r.	Torres et al., 2016
**16**	7.02*^a^*	n.r.	n.r.	n.r.	n.r.	n.r.	3.51*^a^*	n.r.	Samanta et al., 2013
**Gold(III)**									
**21**	3.125*^b^*	3.125*^b^*	n.r.	n.r.	n.r.	n.r.	n.r.	n.r.	Pintus et al., 2017
**28**	0.5–2	n.r.	n.r.	32	n.r.	16–32	32–64	4–16	Soto et al., 2019

*n.r., Not reported; ^a^Minimum Biofilm Eradication Concentration; ^b^Minimum concentration to interfere in biofilm formation.*

The most known and studied gold(I) complex is auranofin [triethylphosphine (2,3,4,6-tetra-*O*-acetyl-β-1-D-thiopyranosato-S)Au(I)], a mixed ligand gold compound in clinical use since 1985 for the treatment of severe rheumatoid arthritis (Shaw, 1999), was first described recently as an antimicrobial agent (Cassetta et al., 2014). Auranofin possesses potent microbicidal effects *in vitro* against *Staphylococcus spp.* and *Candida spp.* (Fuchs et al., 2016; Wiederhold et al., 2017) and also moderate activity against *Staphylococcus aureus* and *Pseudomonas aeruginosa* biofilms (Torres et al., 2016, 2018). Auranofin (**1**) consists of a gold(I) center linearly coordinated to a triethylphosphine and a thiosugar ligand as shown in Figure 1 (Roder and Thomson, 2015). Subsequently, a relatively large number of linear phosphine gold(I) complexes, with a sulfur atom in the coordination sphere, have been synthesized and evaluated for their antibacterial effects, exhibiting a different spectrum of *in vitro* activity against several strains of Gram-positive and Gram-negative bacteria. For example, complexes **2** and **3** were comparably active as complex **1** against Gram-positive bacteria and were more active against *Escherichia coli* (Figure 1). When compared to the reference clinical antibiotics piperacillin and chloramphenicol, gold(I) complexes were found to be less active against Gram-negative bacteria but more active against Gram-positive strains (Novelli et al., 1999). It has been reported that this compound is unable to generate resistant mutants in Gram-positive bacteria. The hypothesis for explaining this fact is that auranofin has multiple targets or an unspecified mechanism of action (Thangamani et al., 2016). Thus, complex **1** causes a reduction in the cell wall and DNA biosynthesis pathway and also in bacterial protein synthesis. In addition, a decrease in the production of important toxins, such as hemolysin and Panton Valentine toxins, occurs when the bacteria are exposed to this compound (Thangamani et al., 2016).

Antifungal activity of complexes **2** and **3** against strains of *C. albicans* and *A. niger* was also determined (Novelli et al., 1999). Lipophilic compound **2** displayed excellent activity against both fungi, being comparable with miconazole nitrate. On contrary, more hydrophilic compound **3** was significantly less active on fungi but was more active than **2** on *E*. *coli*.

Other linear gold(I)–triphenylphosphine complexes displaying interesting antimicrobial profiles are gold(I)–triphenylphosphine complexes containing nitrogen-donor heterocyclic ligands. Thus, Nomiya et al. prepared complexes **4** and **5**, which displayed selective and effective antimicrobial activities against two Gram-positive bacteria (*Bacillus subtilis* and *S. aureus*) and modest activities against one yeast (*C. albicans*). In contrast, poor activities observed in the corresponding silver(I) complexes (Nomiya et al., 2000). The mechanism of antimicrobial action of these heterocycle-containing gold(I)– complexes was not investigated in depth but, according to the authors, it is likely quite different from that of the corresponding silver(I) complexes.

Fillat et al. (2011) described a new family of (aminophosphane)gold(I) thiolate complexes **6** and **7,** also belonging to the type of linear gold(I)–phosphine complexes, which exhibit powerful anti-bacterial activity. As expected, these linear gold(I) complexes are more efficient against Gram-positive microorganisms, presenting excellent activity against *Enterococcus faecalis* and *S. aureus*.

As water is the biologically most relevant solvent, the development of water-soluble gold-based metallodrugs is a matter of increasing interest (Hristova et al., 2013). In this regard, Elie et al. (2009) described water-soluble linear gold(I)– complexes **8** and **9** containing sulfonated phosphanes, which display moderate to high antibacterial activity and moderate antifungal activity. A relevant advantage of complexes **8** and **9** in comparison with the previously reported linear gold(I)– phosphine complexes is their lower hydrophobicity, enabling the preparation of aqueous solutions without the addition of organic co-solvents such as dimethyl sulfoxide (DMSO).

Frik et al. (2012) reported heterometallic gold-silver complexes **10** and **11** and compared their antimicrobial activity to silver salts. The Au_2_Ag complexes resulted up to 10 times more toxic than the silver salts for all Gram-positive (*B. cereus*, *S. aureus*) and Gram-negative bacteria (*E. coli, Salmonella typhimurium*), confirming that the biological activity of these complexes did not originate from the dissociation of the silver perchlorate or triflate from the heterometallic derivatives. The heterometallic compounds were also more toxic to Gram-negative bacteria than the corresponding dinuclear gold complexes, pointing to a synergistic or cooperative effect between the gold and silver metals. This result is of considerable importance for the development of novel metalloantibiotics which may be toxic for both Gram-negative as well as Gram-positive bacteria. For complex **11** containing ClO_4_^–^ contraion, a remarkably high toxicity for Gram-positive bacteria *B. cereus* was observed. Both complexes **10** and **11** were inactive against yeast *S. cerevisiae*.

Barreiro et al. (2012) described several gold(I) sulfanylcarboxylates **12** showing significant activity against *S. aureus* and *Bacillus subtilis*. Similar to other gold(I) compounds, these complexes show good activity against Gram-positive strains (*S. aureus* and *B. subtilis*), but low sensitivity against Gram-positive microorganisms (*E. coli* and *P. aeruginosa*) and fungi (*C. albicans*). The silver complexes incorporating the same ligands were also synthetized, enabling comparison of the activity of silver and gold complexes. Gold compounds displayed better activity against the two Gram-positive bacteria, while the silver complexes generally showed better minimal inhibitory concentration (MIC) values against Gram-negative strains and yeast *C. albicans*. As previously suggested by Nomiya et al. (2000) a different mechanism of antimicrobial action for silver and gold complexes was also hypothesized in which the antibacterial activity of silver(I) complexes is related to the ease with which they undergo ligand metathesis. However, this process would not explain the activity of the related gold(I) complexes.

N-Heterocyclic carbenes (NHC) are extremely good σ-donor ligands, thus forming strong Au-C bonds and constructing Au-NHC complexes that are stable in the presence of biologically relevant thiol groups. Accordingly, for several years, there has been a growing interest in the biomedical applications of gold complexes based on NHC ligands (Mora et al., 2019). Thus, in 2004, Özdemir et al. (2004) reported a novel family of NHC gold(I) complexes of the type [Au(NHC)_2_]^+^.

Among them, *p*-methoxybenzyl derivative **13** was found to inhibit the growth of *P. aeruginosa, S. epidermidis, S. aureus*, and *E. faecalis* with MIC values of 3.12 μg mL^–1^, 6.25 μg mL^–1^, 3.12 μg mL^–1^, and 3.12 μg mL^–1^, respectively. However, complex **13** only showed modest activity against yeast *C. albicans* (Özdemir et al., 2004).

Later in 2007, Ray et al. (2007) reported the Au(I) complexes of 1-benzyl-3-*tert*-butylimidazol-2-ylidene, as well as the Pd and Ag counterparts. While the palladium complexes displayed potent anticancer activity, the gold and silver complexes inhibited the growth of Gram-positive *B. subtilis* but did not show any activity against Gram-negative *E. coli* strains. Specifically, complex **14** showed a twofold stronger antibacterial activity toward *B. subtilis* compared to the corresponding silver complex. Incubation of *B. subtilis* cells with the gold compound increased the cell length, indicating that complex **14** inhibits bacterial proliferation by blocking cytokinesis. In contrast, incubation of *B. subtilis* with the equivalent silver complex resulted in only a very small increase in cell length, which agrees with a different mechanism of antimicrobial action for silver and gold complexes.

N-heterocyclic carbene complex **15** inhibited *E. faecium* and two methicillin-resistant *S. aureus* (MRSA) strains at MIC values of 3.12, 0.64, and 0.64 μM, respectively, being more active than **1** (MICs of 18.40, 2.30, and 2.30 μM) (Schmidt et al., 2017). Complex **14** displayed effective inhibition of mammalian and bacterial thioredoxin reductases (TrxR). As the TrxR system is essential for the antioxidant system of Gram-positive bacteria, this result is in accordance with the particularly high activity observed against Gram-positive strains. TrxR was also hypothesized as one of the multiple bacterial targets of complex **1** (Thangamani et al., 2016), confirming the interest in bacterial Trx and TrxR as viable antibacterial drug targets for gold-based anti-infective agents (Felix et al., 2021).

**FIGURE 2:**
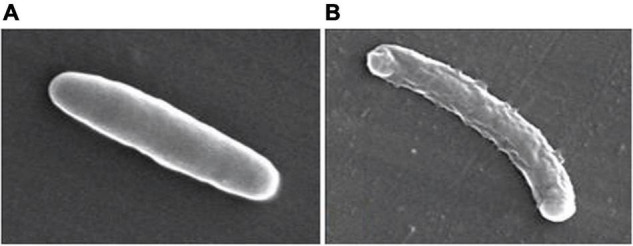
Control *P. aeruginosa* cells **(A)** and cells treated with complex **16 (B)**. Reproduced from Samanta et al. (2013) (CC BY-NC 4.0).

Samanta et al. (2013) described a *N*,*N*’-olefin functionalized bis-imidazolium gold(I) salt **16** of interest for the development of antimicrobial therapies in patients with keratitis-associated eye infections caused by multidrug-resistant bacteria and fungi. Complex **16** was more active against *P. aeruginosa* and *S. aureus* than antibiotics ceftazidime, vancomycin, and piperacilline and more active against filamentous fungi *C. albicans* and *A. fumigatus* than amphotericin B, fluconazole, and voriconazol. The bacterial killing activity was concentration-dependent, and no cell growth was seen even using complex **16** concentrations of 0.5 and 2.5 μM for *P. aeruginosa* and *S. aureus*, respectively. In addition, this Au(I) compound was able to eradicate biofilm formed over contact lenses. In order to better understand the mechanisms of action, interactions between the bacterial cell and complex **16** were observed using a scanning electron microscope (SEM). The images produced (Figure 2) clearly showed a morphological change in the microbial envelope after treatment with complex **16**, which evidenced that the target would be on cell membranes or cell walls. The high binding affinities to bacterial cell membranes displayed by complex **16** in docking studies point to a mechanism of action based on the destabilization of the bacterial membrane, causing loss of structural integrity and a change in bacterial shape. Samanta et al. (2013) hypothesized that the destabilizing effect on the bacterial membrane is caused by the insertion of multiple molecules of complex **16** intercalating the lipids on the membrane.

In summary, several Au(I) complexes tested so far display antibacterial effects with a marked preference for Gram-positive bacteria. This selectiveness toward Gram-positive pathogens could be explained in that: (i) gold(I) complexes inhibit the thioredoxin reductase enzyme and, in Gram-negative bacteria this is compensated by the presence of the glutathione system; and (ii) the permeability barrier conferred by the outer membrane of Gram-negative bacteria impairs the transfer of the complex to the cytoplasm. In addition, other possible mechanisms of action proposed include the disruption of the bacterial cell wall and cytosolic membrane. SEM images of Gram-positive bacteria submitted to gold(I) compounds revealed morphological changes on the bacterial surface and cytosolic leakage, probably due to destabilization of the peptidoglycan membrane by the insertion of these compounds in the lipid bilayer, inducing a loss of the structural integrity of the bacteria.

## Gold(III) Complexes

**FIGURE 3:**
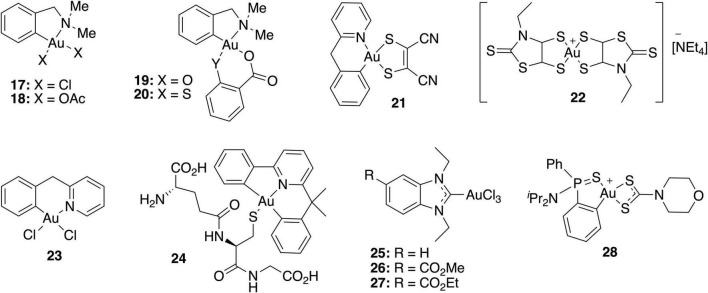
Most representative gold(III) complexes with antimicrobial activity.

Even though gold(III) complexes have been less studied as antibacterial agents than their gold(I) counterparts, the threat of multiresistant bacteria has led to the evaluation of these classical anticancer metallodrugs also as antibacterials. A selection of gold(III) complexes with antimicrobial activity and their chemical structure is shown in Figure 3, as well as a compilation of their antibacterial (Table 4), antifungal (Table 5) and antibiofilm activities (Table 3).

**TABLE 4:** Antibacterial activity (MIC) of the gold(III) complexes.

Compound	MIC (μ g/mL)								References
	Gram-positive					Gram-negative			
	*S. aureus*	*S. haemolyticus*	*B. subtilis*	*K. pneumoniae*	*E. faecium*	*E. coli*	*P. aeruginosa*	*A. baumannii*	
**17**	1–2.5	1–2.5	n.r.	10–25	n.r.	10–25	10–25	n.r.	Parish, 1999
**18**	0.25–1	1–2.5	n.r.	n.r.	n.r.	2.5–10	50–100	n.r.	Parish et al., 1996
**19**	n.r.	n.r.	10*^a^*	n.r.	n.r.	4*^a^*	4*^a^*	n.r.	Dinger and Henderson, 1998
**20**	n.r.	n.r.	12*^a^*	n.r.	n.r.	8*^a^*	8*^a^*	n.r.	Dinger and Henderson, 1998
**21**	3.13	1.56	n.r.	n.r.	n.r.	n.r.	n.r.	n.r.	Pintus et al., 2017
**22**	10.14	n.r.	n.r.	n.r.	n.r.	>100	n.r.	n.r.	Fontinha et al., 2020
**23**	1.36–2.72	n.r.	2.72–5.44	n.r.	11–25	25–50	n.r.	n.r.	Chakraborty et al., 2021
**24**	3.12–6.25	n.r.	3.12–6.25	n.r.	>50	>50	n.r.	n.r.	Chakraborty et al., 2021
**25**	0.38	n.r.	n.r.	8.9	0.57	18.8	40.6	4.0	Büssing et al., 2021
**26**	1.18	n.r.	n.r.	49.2	6.2	>16	40.1	9.2	Büssing et al., 2021
**27**	1.32	n.r.	n.r.	36.7	3.1	27.5	>16	9.2	Büssing et al., 2021
**28**	0.04–0.37	n.r.	n.r.	n.r.	n.r.	5.9–11.8	11.8	0.74	Soto et al., 2019

*n.r., Not reported; ^a^Minimum concentration to create a clearing zone of 0.5 cm: 1, 10, 100.*

**TABLE 5:** Antifungal activity (MIC) of the gold(III) complexes.

Compound	MIC (μ g/mL)							
	*C. albicans*	*C. neoformans*	*C. glabrata*	*C. krusei*	*A. fumigatus*	*A. niger*	*C. resinae*	References
**17**	10–25	2.5–100	2.5–10	10–25	2.5–10	10–25	n.r.*^a^*	Parish, 1999
**18**	n.r.	n.r.	n.r.	n.r.	n.r.	n.r.	n.r.	Parish et al., 1996
**19**	6*^a^*	n.r.	n.r.	n.r.	n.r.	n.r.	4*^a^*	Dinger and Henderson, 1998
**20**	8*^a^*	n.r.	n.r.	n.r.	n.r.	n.r.	1*^a^*	Dinger and Henderson, 1998
**21**	n.r.	>1000	>1000	>1000	n.r.	n.r.	n.r.	Pintus et al., 2017
**22**	12.1	n.r.	9.7	n.r.	n.r.	n.r.	n.r.	Fontinha et al., 2020
**23**	n.r.	n.r.	n.r.	n.r.	n.r.	n.r.	n.r.	Chakraborty et al., 2021
**24**	n.r.	n.r.	n.r.	n.r.	n.r.	n.r.	n.r.	Chakraborty et al., 2021
**25**	n.r.	n.r.	n.r.	n.r.	n.r.	n.r.	n.r.	Büssing et al., 2021
**26**	n.r.	n.r.	n.r.	n.r.	n.r.	n.r.	n.r.	Büssing et al., 2021
**27**	n.r.	n.r.	n.r.	n.r.	n.r.	n.r.	n.r.	Büssing et al., 2021
**28**	n.r.	n.r.	n.r.	n.r.	n.r.	n.r.	n.r.	Soto et al., 2019

*n.r., Not reported; ^a^Minimum concentration to create a clearing zone of 0.5 cm: 1, 10, 100.*

The stabilization of the oxidation state +3 is of great importance to the biological activity of these compounds. Otherwise, the metal center can ultimately undergo a reduction process leading to the formation of Au(0) (Pacheco et al., 2009). Thus, the most common gold(III) complexes used as antibiotics are cyclometallated organometallic gold compounds with the general formula [AuX_2_(L)], in which L is a bifunctional ligand that forms a C-Au bond, as well as a coordinate N-Au bond. The two remaining positions at the square planar gold(III) center are usually occupied by two monodentate or one bidentate anion, leading to the formation of neutral complexes (Tiekink, 2002).

Parish et al. performed a variety of biological tests to evaluate [AuCl_2_(damp)] (**17**) for antimicrobial activity; the complex displayed moderate, broad-spectrum activity against bacteria and fungi, but was less active than the control drugs ciprofloxacin and amphotericin B (Parish, 1999). In order to improve the antibacterial activity of complex **17**, both chloride atoms were replaced by acetate ligands, leading to water-soluble complex **18** (Parish et al., 1996). Similar to its chloride analog, complex **18** showed a broad spectrum of antibacterial activity, with some preference to Gram-positive *S. aureus* and *E. faecalis*. The MIC values of complex **18** against Gram-positive bacteria were of a lower order of magnitude than the cytotoxic concentrations against Chinese hamster ovary cells, indicating *in vitro* selectivity for these microorganisms. On the other hand, gold(III) complexes **19** and **20**, containing chelating thiosalicylate and salicylate anions, respectively, showed significantly high activity against bacteria *E. coli*, *B. subtilis*, and *P. aeruginosa*, and fungi *C. albicans* and *C. resinae* (Dinger and Henderson, 1998).

The gold(III) 1,2-dithiolene cyclometalated complex **21** was tested against a panel of Gram-positive and Gram-negative bacteria and three yeasts of the *Candida* spp., showing remarkable bacteriostatic activity against *S. haemolyticus* and *S. aureus*, with MIC values of 1.56 and 3.13 μg/mL, respectively. However, none of the tested Gram-negative bacteria or fungi were sensitive to this gold(III) complex. The biofilm inhibitory effect if complex **21** was also described in this study against *S. aureus* and *S. haemolyticus*, where they highlight 3.125 μg/mL as the minimum concentration to interfere in biofilm formation. However, due to turbidity of the solution, a concentration that fully inhibits biofilm formation was not provided (Pintus et al., 2017).

The gold(III) bis (dithiolene) complex **22** containing an *N*-ethyl-1,3-thiazoline-2-thiane dithiolate ligand showed significant activity against Gram-positive bacteria *S. aureus* and fungi *C. glabrata* and *C. albicans* but not against Gram-negative *E. coli* (Fontinha et al., 2020). As in the case of gold(I) complexes, several studies support that thioredoxin reductase is a possible target of these complexes. In addition, complex **22** is stable under biological conditions, being an important characteristic if this compound is ever to be considered as a drug.

More recently, Chakraborty et al. (2021) reported several gold(III) complexes with bidentate N^N ligands, cyclometalated bidentate C^N and terdentate C^N^N scaffolds. Even though those cyclometallated Au(III) complexes do not show any antibacterial activity against *E. coli*, complexes **23** and **24** inhibited the growth of *B. subtilis*, *S. aureus*, and MRSA, with MICs ranging from 3.12 to 25 μM.

These compounds do not affect adenosine triphosphate (ATP) synthesis, or membrane potential or permeability. RNA sequencing revealed that these gold(III) complexes upregulated genes related to metal transport, major facilitator superfamily (MFS)-transporter, genes responsible for arsenic metabolism, oxidoreductases, and proteases, among others. However, they downregulated genes related to cell wall formation, contact dependent growth inhibition, and ABR transporter genes (Chakraborty et al., 2021).

In view of the biological interest of Au(I) complexes with NHC ligands, the corresponding gold (III) analogs were also prepared and investigated as organometallic antibacterial drugs. Au(III) NHC complexes **25**–**27** showed higher antibacterial activity against the Gram-positive MRSA and *E. faecium* than against the Gram-negative strains (*A. baumannii*, *E. coli*, *Klebsiella pneumoniae*, and *P. aeruginosa*), as previously reported for the related Au(I) NHC complexes. All target complexes were efficient inhibitors of bacterial TrxR, suggesting that inhibition of this enzyme might be involved in the mechanism of antibacterial activity (Büssing et al., 2021).

The interest of Au(III) dithiocarbamate complexes as antimicrobial agents dates from the 90 s, when the potential activity against *Streptococcus pneumoniae* of a series of Au(III) complexes with dithiocarbamates derived from α-amino acids (DL-alanine, DL-valine, L-valine, and DL-leucine) was reported (Criado et al., 1992).

Since the biological activity is strongly related to the coordination sphere of gold, a good strategy to modulate the biological properties of gold complexes would be to alter the ligands attached to the metallic center. This strategy has been widely applied to the development of gold-based anticancer drugs. Bertrand et al. (2015) studied cyclometalated Au(III) complexes with 1,3,5-triazaphosphaadamantane (PTA) and thio-α-D-glucose tetraacetate (GluS) ligands, chosen on account of the properties that could be conferred to the gold complexes. Thus, the PTA ligand was chosen for its good water solubility. As for the GluS ligand, Au(III) binding to thiolates would avoid ligand exchange reactions with biological nucleophiles that would lead to inactivation of the compound. In addition, the presence of the sugar unit would have important implications in molecular recognition phenomena.

Considering these previous studies, the effect of different dithiocarbamate ligands on the therapeutic index of dithiophenylphosphine Au(III) complexes has been extensively investigated (Soto et al., 2019). Thus, a group of (S^C)-cyclometallated gold(III) complexes containing a 1,1-dithio ligand was developed and tested against a wide panel of Gram-positive and Gram-negative bacteria. Among these, complex **28** not only exhibited antibacterial *in vitro* activity against multidrug resistant microorganisms, especially *S. aureus*, *S. maltophilia*, *P. aeruginosa*, and *H. influenzae*, but also displayed low cytotoxicity. Complex **28** was able to in inhibit biofilm formation by its addition before the adhesion phase at low concentrations in *S. aureus* strains. The minimum biofilm inhibitory concentration (MBIC) values ranged between 0.5 to 2 mg/L for both MRSA and MSSA, only two-fold higher than their MIC in planktonic phase. For Gram-negative bacteria, a similar trend was observed for biofilm forming strains of *P. aeruginosa, K. pneumoniae*, and *A. baumannii*, which showed MBICs two-fold higher than their corresponding MIC. In addition, **28** was also highly stable under physiological conditions and is expected to maintain its antibacterial activity *in vivo* in the same way.

In summary, albeit less studied than their Au(I) counterparts, Au(III) complexes exhibit potent antibacterial activities and do not generate bacterial resistance. However, very little attention has been given to the elucidation of their modes of action. The data available indicate that, opposed to Au(I) complexes, Au(III) complexes may not have any effect on reactive oxygen species production, membrane permeability or membrane depolarization (Chakraborty et al., 2021). The low probability of development of mutations also suggest multiple targets for the bactericidal action of Au(III) complexes (Soto et al., 2019). In-depth mechanistic studies are still required to fully elucidate the mode of bactericidal action of Au(III) complexes.

## Conclusion

Increasing resistance to antibiotics is one of the more pressing problems in modern medicine. To tackle this, researchers soon turned their attention to gold. During the past decade, gold-based drugs have become an interesting topic in pharmaceutical research and metallodrug chemistry because many of these drugs have shown potent activity against several bacterial strains, including both Gram-positive and -negative organisms. Unfortunately, promising *in vitro* results are not always correlated with significant *in vivo* effects. Despite all the efforts, scientists have struggled to develop a practical gold-based antimicrobial therapy.

One of the fundamental reasons for this is the complicated biological chemistry of gold compounds; the ligand exchange behavior of the complexes in gold drugs and the Au(III)/Au(I) redox chemistry must be considered, in addition to the problems of hydrolysis and protein binding. In this regard, future developments in the field of gold-based antibiotics require more knowledge about the mechanisms of their *in vivo* distribution and/or degradation.

Gold complexes have unique modes of action, which is a plausible explanation for the observed lack of detectable resistance. However, as the mechanisms of antimicrobial metal toxicity remain uncertain, the identification of bacterial targets and uptake pathways are key issues for promoting the clinical application of gold-based antibiotics.

To overcome the present disadvantages of gold-based drug candidates, several strategies are currently being investigated. A new trend, which has also entered the pharmaceutical chemistry of gold complexes, is the use of encapsulating techniques. Accordingly, the biodistribution of gold antibiotics may be improved by inclusion in cyclodextrins (Morgen et al., 2019; Tomasello et al., 2021). Recent studies have also described a potent synergistic microbicidal effect with antibiotics, suggesting that gold compounds may have great application prospects in combination therapy, potentiating the efficacy of clinically used antibiotics (Ratia et al., 2022).

There is clearly much untapped potential of the use of gold complexes for antimicrobial applications. However, for these drug candidates to be effectively and safely used, more research is required. Better understanding of the structure, function, and mechanisms that give gold-based agents higher antibacterial activity and lower toxicity will lead to the design of better gold complexes that are safe and effective for systemic administration. As the chemical properties of gold complexes can be fine-tuned by proper ligand design (Storr et al., 2006), new generations of various gold-based antibiotics that exhibit more effective antibacterial activities can be foreseen.

To conclude, the future is bright for gold-based antibiotics and the coming years will surely see further advances in this exciting research field. Hopefully, a number of gold-based antibiotic drug candidates will be able to reach clinical trials and the market within the next decade and thereby become a solution for the treatment of bacterial infections resistant to antibiotics.

## Author Contributions

SS: conceptualization. RS and SS: writing-original draft preparation. CR, RS, and SS: writing—review and editing. All authors have read and agreed to the published version of the manuscript.

## Conflict of Interest

The authors declare that the research was conducted in the absence of any commercial or financial relationships that could be construed as a potential conflict of interest.

## Publisher’s Note

All claims expressed in this article are solely those of the authors and do not necessarily represent those of their affiliated organizations, or those of the publisher, the editors and the reviewers. Any product that may be evaluated in this article, or claim that may be made by its manufacturer, is not guaranteed or endorsed by the publisher.
